# Effect of video tutorial delivery method on D/HH students’ content comprehension

**DOI:** 10.3389/fpsyg.2022.872946

**Published:** 2022-11-29

**Authors:** Khalid M. Almalhy

**Affiliations:** Curriculum and Instruction Department, King Saud University, Riyadh, Saudi Arabia

**Keywords:** deaf and hard of hearing students, multimedia, captioned text, sign language interpreter, video tutorials, deaf education, Saudi Arabia Sign Language

## Abstract

**Introduction:**

Using video tutorials to deliver instructional content has become common practices nowadays. However, it is required to investigate how to implement new methods to deliver instructional content to deaf students to ensure success of their learning and reduce their reliance on personnel support or consultation from hearing peers. Therefore, and in light of cognitive theory of multimedia learning, this study experimented three different video tutorial methods to deliver instructional content that are tailored to deaf students’ learning needs. The three methods included: (a) sign language only, (b) captioned text only, and (c) sign language and captioned text combined.

**Methods:**

The study applied a mixed methods research design using pretest-posttest quasi-experimental design (tests scores) and qualitative research design (interviews). Fifty-four undergraduate deaf students from a large university in Saudi Arabia participated in this study, and of those participants, fifteen deaf students participated in semistructured interviews.

**Results and discussion:**

One-way analysis of variance results showed that using video tutorial that presents declarative content with captions only was significantly effective in comparison with the other methods. While the video tutorial that presents procedural content with sign language only was significantly effective in comparison with the other methods. Interview results confirmed the quantitative results. Practical and theoretical implications are discussed.

## Introduction

### Background

In the recent past, technologies have helped transform educational systems through the impressive progress of information and communication technologies (ICT). Indeed, the availability and usability of these instructional technologies have improved accessibility to and utilization of various content for people with disabilities (Debevc et al., 2015). According to Al-Ibrahim (2019) and Mirghani (2020), the predominant pedagogy in Saudi Arabia across all subjects at all levels of education emphasizes direct instruction with memorization and didactic rote learning. However, since the inception of educational reforms relative to the integration of ICT in secondary schools in 2007, national policies known as the King Abdullah bin Abdul-Aziz Project for Public Education Development (*Tatweer* in Arabic) have recommended inquiry strategies and facilitating critical thinking in pedagogical practices. This nationwide initiative supports ICT integration in education and using innovative teaching practices in all subjects (Alenzi, 2015; Allmnakrah and Evers, 2020).

Research has revealed significantly lower levels of educational achievement for deaf students (Topal et al., 2017). Although curricula are usually designed with equality of access for all learners in mind, deaf students still struggle due to insufficient learning resources and the use of instructional strategies not specifically designed for them (Vinoth and Nirmala, 2017; Hashim and Tasir, 2020). Appropriate instructional tools and technical support to strengthen deaf students’ learning environment could improve their learning processes, interaction skills, and academic performance (Hashim and Tasir, 2020). With rapid advances in digital technology, deaf students can access appropriate learning technologies to facilitate the acquisition curricular content essential for their academic and economic achievement (Abuzinadah et al., 2017).

Digital technologies play an important role in education and researchers in all fields have increasingly recognized them. Thus, to help prepare students for future jobs, instruction about digital technology should be considered for all types of students (Zhang, 2014; Abuzinadah et al., 2017). However, research on instructing deaf students about digital technology in Saudi Arabia is still in an early stage, with only a few studies having been conducted (e.g., Zhang, 2014; Abuzinadah et al., 2017).

A few studies have investigated how to instruct deaf students in using digital technology to teach them computer programming from two perspectives. First, research has explored whether deaf students were interested in studying computer programming (Abuzinadah et al., 2017), and second, research has examined the effect of using digital technology to enhance deaf students’ skills in problem solving (Topal et al., 2017). Findings from these studies revealed that although the deaf students who learned to program computers acquired programming skills to contribute to the technology sector in Saudi Arabia, teaching students to program required two types of instruction: (a) teaching declarative knowledge (e.g., definitions, concepts, facts, and terminologies) and (b) teaching procedural knowledge (e.g., the steps for turning on an electronic device; Abu-Zaid and Khan, 2013; Zhang, 2014).

In general, deaf students in Saudi Arabia have little or no access to on-screen written texts and video subtitles in technological materials (El Ghoul and Jemni, 2009; Almotiri, 2017; Topal et al., 2017). These circumstances have encouraged special education experts to seek effective solutions, and they have found that multimedia can be highly effective for deaf students (El Ghoul and Jemni, 2009; Greg, 2014). Video-based instruction is one of the best-known mediums for conveying content and is especially beneficial for deaf students (El Ghoul and Jemni, 2009; Topal et al., 2017; Hashim and Tasir, 2020).

### Problem statement

Only limited online content in the Arabic language targets deaf students, especially in the area of teaching them how to use digital technology (Alasim, 2020a), which requires they acquire both procedural and declarative knowledge. In most cases, both types of knowledge require designing online materials to demonstrate the concepts in digital technology; however, research in teaching deaf students to use digital technology is still in an early stage (Zhang, 2014; Abuzinadah et al., 2017; Topal et al., 2017).

To investigate video design that ensures improved practices in teaching deaf students how to use digital technology, video-based instruction was chosen as an intervention in this study. No current guideline (e.g., the Web Content Accessibility Guidelines) state whether or how captions should be included in a video with a sign language interpreter. Therefore, to determine the optimal video tutorial delivery methods to instruct deaf students about digital technology topics, this study aimed to investigate, for both declarative and procedural knowledge, the effects of the following methods: (a) video with a sign language interpreter alone (S); (b) video with captions alone (C); and (c) video with both a sign language interpreter and captions (S&C). Hence, two research question guide this study.

Research question 1: Do the illustration methods of a sign language interpreter alone, captions alone, or both together differ significantly for increasing deaf students’ acquisition of declarative knowledge?

Research question 2: Do the illustration methods of a sign language interpreter alone, captions alone, or both together differ significantly for increasing deaf students’ acquisition of procedural knowledge?

## Literature review

The American Speech-Language-Hearing Association (2022), which based its latest structure on the recommendations of John Greer Clark, a professor of audiology, categorizes hearing impairment according to five levels. The ranges indicate the number of decibels at which a sound must be for the person with hearing impairment to be able to hear it: (a) Level 1 or Mild: 26–40 dB; (b) Level 2 or Moderate: 41–55 dB; (c) Level 3 or Moderately Severe: 56–70 dB; (d) Level 4 or Severe: 71–90 dB; and (e) Level 5 or Profound: 91+ dB (American Speech-Language-Hearing Association, 2022).

### Education of deaf students in Saudi Arabia

Until 1990, deaf and hard of hearing (D/HH) students in Saudi Arabia learned in private schools, such as the Al-Amal Institute for the Deaf (Abu Shaira, 2013). Currently, the education system in the country offers D/HH students at the elementary and secondary levels two general placement options: (a) self-contained classrooms for students whose hearing loss ranges from 21 to 69 dB, which involve instruction using oral language designed for those D/HH students who have the ability to hear their teachers; and (b) special schools or self-contained classes in regular schools for deaf students whose hearing loss is 71 dB or greater and who use sign language. For those who use sign language, the type employed includes a combination of signs from Saudi Arabia Sign Language (SASL) and Arab Sign (AS), which is used in multiple countries throughout the Middle East (Alasim, 2020b). SASL, as the official language of the Saudi deaf community, has challenges regarding Saudi teachers’ lack of knowledge and fluency in the use of this naturally generated sign language that was developed within the country and that is used by members of the Deaf community in Saudi Arabia. In contrast, teachers have greater familiarity with and ease of using AS, which is as a collection of invented signs upon which members of a committee composed of individuals from various Arabic countries have agreed (Alamari, 2017).

When deaf students learn along with their hearing peers in academic institutions and vocational programs, they demonstrate significant gaps in the knowledge and literacy skills essential for success in higher-level education (Alshawabkeh et al., 2021). For instance, El Ghoul and Jemni (2009) found that deaf students exhibited low levels of reading and memorization compared with hearing students, although they had learned in the same classroom with the same teachers; other researchers have reached the same findings (Laabidi et al., 2014; Al Zaylai, 2019; Alasim, 2020b). Note that although there some research has shown a correlation between disability of deafness and certain learning/cognitive disorders (Edwards, 2012), when hearing instructors and deaf students are more familiar with different sign languages—in this case AS vs. SASL, or educators are not familiar with any sign language at all, poor communication between the two groups factors into deaf students’ lower achievement rates (Anderson and Reilly, 2020).

In 2011, a large university in Saudi Arabia initiated a deaf program and began to accept deaf students in undergraduate academic programs; the university is now considered a pioneer in the Middle East for taking such a step (Al-Ibrahim, 2019; King Saud University, 2020). The program established special admissions requirements for all deaf students, including a special admissions exam and an interview that ensured the applicants’ ability to master several skills, including SASL and reading/writing proficiency. Since then, researchers have sought to identify the best instructional methods for these students (Abuzinadah et al., 2017). For deaf students who had limited exposure to SASL during their elementary and high school years, deaf students who acquired at least some facility in AS, and those students who may use a combination of both sign systems, the complexity and rigorousness of the content in postsecondary academic and vocational curricula pose challenges for programs when working to accommodate the diversity of deaf students’ communications, reading and learning preferences, and needs (Hussein, 2010).

### Video-based instruction

The advent of video-based instruction has presented new opportunities for educators to design rich learning environments both in the traditional face-to-face setting and in online learning. The combination of more than two mediums—image, sound, and text—results in an effective learning method; using video tutorial increases student interaction and makes teaching more efficient (Mayer, 2009; Yoon and Kim, 2011). However, embedding video in multimedia teaching material is critical to improve instructional environments (Ljubojevic et al., 2014; Martins et al., 2015).

In the Saudi deaf community, deaf students who rely on sign language as their primary form of communication may encounter many challenges with language (El Ghoul and Jemni, 2009; Almotiri, 2017). Video-based instruction could help these students counter those challenges and develop their language skills (Napier et al., 2018). Moreover, video tutorial can enable deaf students to access content *via* a sign language interpreter or captioning, with the added advantage of being able to repeat or replay the content as many times as necessary until it is mastered before applying it (El Ghoul and Jemni, 2009). In addition, educators have been making recent efforts to increase the availability of online content with captioning and other video delivery methods for deaf students. Despite these prospects, clear instructional techniques and guidelines for expanding video technology usage to accommodate the deaf students’ requirements are limited (Mehta et al., 2019).

The Web Content Accessibility Guidelines 3.0 (Panda and Chakravarty, 2020), which the Web Accessibility Initiative publishes, provides advice on accessibility for developing multimedia material, such as animation and video. However, this advice is general and does not define whether captions should be included in a sign language-interpreted video. The guidelines state that deaf persons need captions or sign language interpretation, but it does not mention where and how these should be incorporated into multimedia (Panda and Chakravarty, 2020).

#### Captions

Captions are verbatim text (subtitles) shown while visual content is displayed. Thus, captioning transforms spoken words into on-screen text. This text is either open captioning or closed captioning. Open captions cannot be turned off and are always displayed on the screen; whereas users can control closed captioning (Shepherd and Alpert, 2015). Captions can be created before a video is displayed (offline captions), which is the case with educational videos for secondary and postsecondary students (Stinson and Stevenson, 2013; Alsalamah, 2020). Adding captions to video clips could lead to a universally beneficial design contributing to accessibility for individuals with and without disabilities, including deaf students (Butler, 2019). Other researchers have found that deaf students could comprehend lecture content more effectively when additional content and vocabulary definitions accompanied the expanded captions vs. when lectures were presented only verbally (Szarkowska et al., 2011; Cawthorn and Leppo, 2013; Stinson and Stevenson, 2013). In a recent systematic meta-analysis of experimental studies of captioning to support deaf students in higher education, Alsalamah (2020) reviewed seven studies. Four of the studies used real-time captioning and three used offline captioning. Overall, the use of both real-time and offline captioning facilitated postsecondary-level deaf students’ comprehension and retention of science content. In addition, Yoon and Kim’s (2011) findings indicated that deaf students benefitted from displaying content through both captions and signing. Research has found that using captions modified to accommodate the lower reading comprehension skills typical among deaf students in combination with supplemental instructions from an instructor can increase students’ comprehension and retention of academic content. Thus, captioning is important for deaf students, but to be optimally effective in facilitating their acquisition of academic content, the students’ reading level, their prior familiarity with the content, and the type of content presented must be considered when determining deaf students’ content comprehension (Debevc et al., 2015; Al-Ibrahim, 2019).

Research also has found that altering the text of the broadcast television programs’ captions by simplifying vocabulary, using bold font and underlining, reducing subtitle speed, and using simple syntactic structure increased 8 to 13 year-old deaf students’ comprehension of commercially broadcast video content (Tamayo and Chaume, 2017). Romero-Fresco (2016) proposed a model for assuring the quality of captioning so that deaf individuals can have better access. This model emphasizes both linguistic and technical parameters that captioners should consider. These include content, grammar, readability, sociocultural aspects of the viewers, speed, formatting, font, and the way the captions appear and disappear. Vanderplank’s (2019) research suggested that caption users’ motivation, engagement, language level, and prior knowledge of content could be supported by interactions with an instructor to guide users in applying the content presented *via* the captions more efficiently.

#### Sign language

Sign languages are *bona fide* visual gestural languages that use hand configurations, hand location, hand movements, facial expressions, lip patterns, and body language to convey content and express emotions (Cormier et al., 2010). Similar to spoken languages, sign languages vary among countries and have their individual vocabularies, as well as syntactic, semantic, and morphological features. Sign language users express emotions primarily through nonmanual features including eyebrow raising and specific lip movements, such as a downturned lower lip. Signed languages also vary within the same country and they constantly evolve as deaf users introduce new terms and experience new concepts (Mayberry, 2006).

Sign language features and usage are a vast area of research that includes studies on the use of signing in education, psychology, and linguistics. The application of signing in ICT research, especially in e-learning, is a rapidly growing research area (Vinoth and Nirmala, 2017; Hashim and Tasir, 2020). For example, in a recent study, Galindo-Neto et al. (2019) validated an instructional video incorporating signing for teaching deaf students about cardiopulmonary resuscitation. Experts have judged that a video tutorial that includes sign language if a valid form of content, and deaf students found the content comprehensible. Thus, video supplemented with sign language can be an appropriate delivery method for deaf students to acquire procedural content.

The development and application of signing avatars in virtual learning environments for use with deaf students has received considerable emphasis as e-learning designers determine how best to make a signing avatar express the nuances of semantic meaning in conveying specific content (Zirzow, 2015; Smith and Nolan, 2016; Lee et al., 2020). However, designing signing avatars presents challenges in that they must incorporate the fluidity of movement transitions between signs and fingerspelling while simultaneously expressing facial expressions congruent with the content. Lee et al. (2020) further enhanced signing avatars by designing semirealistic human models to include differences in racial and gender features to preserve the contextual accuracy with which the content is presented *via* signing (Green et al., 2022).

#### Combination of sign language and captions

Including captions in combination with a signed video can provide deaf students full access to content *via* the e-learning environment’s design (Yoon and Kim, 2011; Debevc et al., 2015). For example, in a comparison of Slovenian deaf adult users’ comprehension of the content presented in signed interpreted videos with and without captions, Debevc et al. (2015) found that the deaf users increased their content comprehension when the content was presented with both signing and captioning. Their study’s findings corroborated Yoon and Kim’s (2011) research regarding the combination of signing and captions as better facilitating deaf students’ content comprehension.

### Theoretical framework: Cognitive theory of multimedia learning

Several researchers have demonstrated that learning through e-learning multimedia (e.g., signing, captions, graphics, and animations) can enhance deaf students’ learning (Martins et al., 2015; Abuzinadah et al., 2017; Vinoth and Nirmala, 2017). The design of multimedia instructional materials is often based on an information-processing cognitive theory of multimedia learning that incorporates the concept of cognitive overload. This terminology refers to a learning situation in which the learner’s task exceeds the capacity of their cognitive system to process the verbal and visual information efficiently (Mutlu-Bayraktar et al., 2019). Hashim and Tasir (2020) and Mutlu-Bayraktar et al. (2019) advised using strategies to reduce cognitive load in multimedia learning, such as eliminating redundancy, eliminating extraneous content, and synchronizing visual and verbal material. Importantly, Ibrahim et al. (2016) advocated that the potential users’ learning style preferences should be considered in designing e-learning. Lang et al.’s (1999) analysis of deaf college students’ learning styles according to their learning preferences indicated that the students preferred dependent, teacher-centered learning in which the instructor determined and prioritized a course’s content with the instructor providing sample notes and supporting materials, such as outlines. Thus, multiple factors should be considered in designing tutorial videos that will ensure deaf students’ comprehension and application of content.

According to Hong et al. (2018), acquiring cognitive skills (e.g., learning computer programming skills) encompasses two phases: declarative and procedural. As defined Oosterhof et al. (2008) defined it, the declarative phase of knowledge about a skill or concept focuses on an individual acquiring basic information and explanations of principles. In contrast, the procedural knowledge phase involves the individual applying the information gleaned from the declarative knowledge phase. Procedural learning requires using declarative knowledge. According to Ezeamuzie (2022), instructional strategies to teach introductory computer programming should enable a learner to engage in the declarative learning of abstract programming concepts and then the learner should design a computer program to engage in procedural learning by putting into action what has been acquired about the programming concepts. Thus, declarative and procedural knowledge can be used as a framework for investigating ways to design multimedia instruction in computer programming and computer literacy for deaf college students (Hong et al., 2018).

## Methodology

### Participants

This study’s targeted population was undergraduate deaf students enrolled at a large university in Saudi Arabia. As noted above, this population of deaf higher education students must meet certain minimum standards regarding SASL and reading comprehension for admission into the university. I limited the sample to deaf students whose hearing loss was 71 dB or greater (Level 4 or above). I excluded students with a lesser average level of hearing impairment as well as those whose hearing could be augmented with hearing aids because the ability to use such prostheses could have affected the results. Those whose hearing impairment could be offset with such technology were not the target group of this study. For these same reasons, no one with cochlear implants was included in the study sample. *Via* their university emails, I asked 104 deaf students through demographic data forms to participate in the research, and of the 104 invited participants, 72 responded. Through informal initial interviews, a reading test, and another assessment related to SASL familiarity, I assessed the students’ reading comprehension and understanding of sign language. Based on the results, 18 of the 72 were excluded because their reading comprehension was not at the level required to understand the type of content and/or their understanding of SASL was not adequate to benefit fully from the study’s methodology. After confirming all the remaining participants (*n* = 54) had effective sign language and reading skills, the sample was randomly assigned—to reduce the effect factors unequal groups might cause—into three groups of 18 participants each as follows: Group S (Sign only), Group C (Captions only), and Group S&C (Sign + Captions). Table 1 details the participants who completed all the study quizzes and examinations, along with their demographic characteristics. The detailed procedures help to reduce any effect that might have come for the study’s limited number of participants (Howell, 2012). Lastly, participation in this study was voluntary and every participant signed a consent letter prior participation. The Institutional Review Board approved the study prior to data collection.

**Table 1:** Demographic characteristics of participants in the three experimental groups.

Baseline characteristic	Group S (sign language alone)		Group C (captions alone)		Group S&C (sign language with captions)		Full sample	
	*n*	%	*n*	%	*N*	%	*N*	%
**Gender**								
Male	6	33.3	4	22.2	8	44.4	18	33.3
Female	12	66.7	14	77.8	10	55.6	36	66.7
Total	18	100.0	18	100.0	18	100.0	54	100.0
**Academic level**								
Foundation year	13	72.2	15	83.3	15	83.3	43	79.6
First 2 years of university study	5	27.8	3	16.7	3	16.7	11	20.4
Total	18	100.0	18	100.0	18	100.0	54	100.0

Specifically, the research sample included 54 deaf participants who were randomly distributed among the three groups. Of these, 66.7% were female and 33.3% were male. Of the total participant pool, 79.6% were in their foundation year and 20.4% were in their first 2 years of university.

### Research design

**Figure 1:**
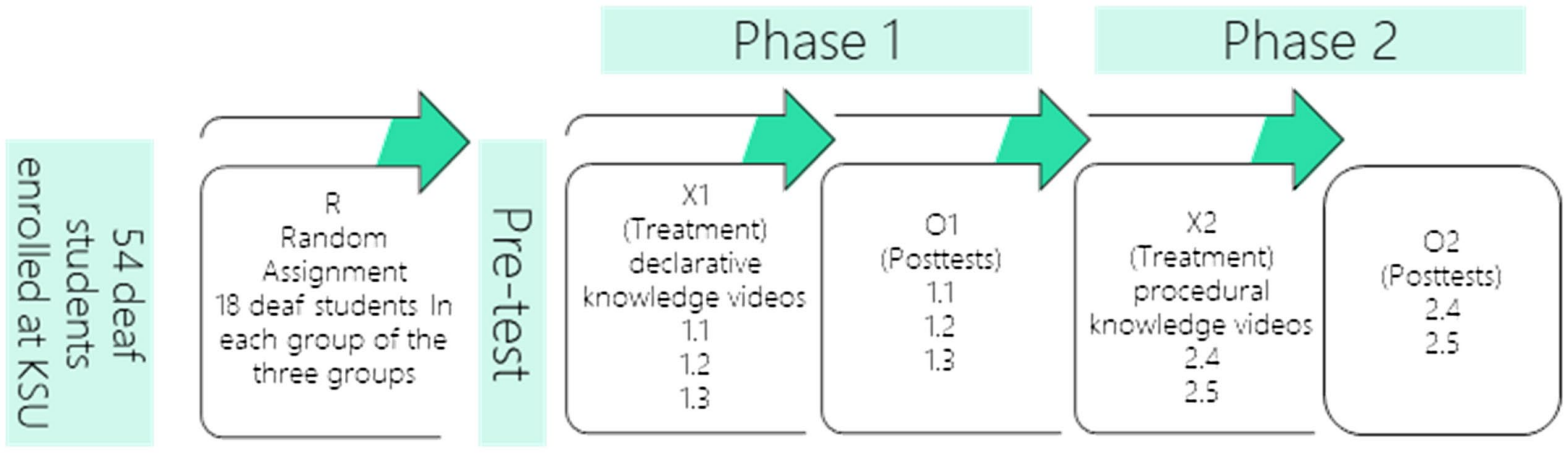
Research design: pre- posttest quasi-experimental.

The study adopted a mixed methods research approach. The quantitative portion of this approach employed a pretest-posttest quasi-experimental design (see Figure 1). In the pretest-posttest design, the deaf students received a pretest before they watched the video tutorials. Then, they received a posttest to evaluate their acquisition of declarative and procedural knowledge after watching video tutorials related to cyberbullying and identity protection. In addition, the qualitative portion of the study employed semistructured interviews to enhance the research’s results and to ascertain the participants’ attitudes regarding the best method of illustration that could be embedded in video tutorials. I depended on commonly designed research in the educational technology field (Rabu, 2015; Jumaat and Tasir, 2016) because it is more convenient, ethical, and practical (Paulus et al., 2014). The mixed methods research approach is useful for gaining deeper insight of the issue that is being investigated compared to a standalone quantitative or qualitative study because it integrates the benefits of both methods (Creswell, 2014). However, the results cannot be generalized; instead, the main objective of this study is to introduce and discover evidence of the best means of illustration that can be embedded within video clips to gain declarative and procedural knowledge of specific technological content, especially in the context of cyberbullying and identity protection for deaf students.

### Materials

#### Instruction and content

**Figure 2:**
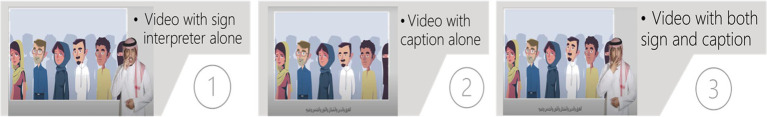
Clips from the three video delivery methods.

Three of the designed videos focused on the acquisition of declarative knowledge and two videos highlighted the acquisition of procedural knowledge. The study followed the general standard for designing such tutorial videos (Mayer, 2009). Thus, all videos were limited to no more than 2 min each and captions were presented at approximately 140 wpm, which is deemed an acceptable speed (Smith et al., 2017; Allman et al., 2019). As Figure 2 shows, the designed videos’ instructions were presented with a sign interpreter alone, with captions alone, and with both sign and captions. Each group watched the five videos with one of the three delivery methods. The content of each of the videos was as follows.

- Video 1: What is cyberbullying?
- Video 2: How to protect yourself from cyberbullying
- Video 3: How to treat yourself after cyberbullying
- Video 4: The process of protecting your devices from hacking
- Video 5: How to activate the two-factor authentication for social network accounts

#### Guidelines

The participants received detailed guidelines in two formats, text-based and sign language-based, to explain the following bulleted items. An official university sign language interpreter, who is employed to help deaf students communicate with faculty members, presented the sign-language-based content in SASL.

- The aim of the study
- How the study would be implemented (in steps)
- Other related information, such as: the importance of the study, the consent form, the number of attempts that would be permitted for taking the study quizzes and exams, the length of the break periods that would be provided during the experiment, and the process the deaf students would need to follow to submit their completed quizzes and exams
- The investigator’s contact information

#### Presurvey

The study also included a presurvey form including demographic information and other research-related questions, such as participants’ academic level and their prior knowledge of ICT subjects, to obtain basic background information on each; this was administered immediately before the experiment.

#### Pretest

To ensure that the study sample had no prior knowledge on cyberbullying and identity protection topics, a pretest was applied. The results showed that none of the participants had prior knowledge of this topic. The pretest was a short survey consisting of Yes/No questions that asked participants about their prior knowledge regarding the topics of the five videos; all the answers provided to all the questions on the pretest were “No.”

#### Posttests

For the posttests, correct responses on each question on the quizzes and on the final examinations were scored as 5 points and incorrect responses were scored as 0 points, with a maximum of 25 points for each quiz in the declarative section; 45 points and 50 points on the first and second quizzes (Quiz 4 and Quiz 5) in the procedural section, respectively; and 50 points for each of the two examinations. All testing items were composed according to established standards regarding deaf students’ reading levels (Napier et al., 2018). This composition was achieved through the following steps: (a) the quiz and exam questions were constructed, (b) experts in the field of deaf education then validated these questions to ensure their fitness for the participants’ reading level, and (c) a sample of five deaf students (who were not part of the study sample) and one assistant professor whose major was deaf education reviewed the questions prior to the study being conducted; this pilot study group reported no difficulties.

In phase 1 of the experiment, three quizzes (one per video; five questions per quiz) tested participants’ declarative knowledge, and a comprehensive final examination consisting of 10 multiple-choice questions measured declarative knowledge (Abu-Zaid and Khan, 2013). Each question had four response options where one was the correct answer and the other three were distractors (see example below).

Q: Cyberbullying is?

(a) Positive behavior

(b) Bad behavior

**(C) Unlawful or criminal behavior**

(d) A form of racism

For phase 2 on procedural knowledge, participants took two quizzes (one per video). For the fourth video, the quiz comprised nine questions, and for the fifth video, the quiz comprised 10 questions. In addition, participants completed a comprehensive final examination of 10 multiple-choice questions appropriate for measuring procedural knowledge (Abu-Zaid and Khan, 2013). Again, each question had four options with one being the correct answer and the other three being distractors (see example below).

Q: Apply the process of setting up a secure password. Then choose the most secure password from the following.

(a) 537**ACfg

(b) aa2321@

(c) AcvAbvf#

(d) 11,223,344

In phase 2, participant responses were scored as stated previously in the description of the scoring for phase 1 (5 points for correct answers; 0 points for incorrect answers), with a maximum of 45 points for the fourth quiz, 50 points for the fifth quiz, and 50 points for the 10-question multiple-choice examination.

### Design framework

#### Video-based instructions design

Before the study, I followed the necessary regulatory procedures for university approval of human research. Next, I chose the topics: The declarative knowledge topic was established as cyberbullying and the procedural knowledge topic was established as information security. Both topics stem from the field of digital technology and participants confirmed never having studied them in previously taken ICT courses. This supported the choice of applying the research design described previously.

Next, a certified design team developed three instructional videos about cyberbullying and two about information security. In these, the images and animations strongly matched the content to facilitate the comprehension process. Experts validated these instructional videos in two stages. For the first stage, assistant professors who specialize in educational technology and computer education validated the videos. Next, three copies of each of the five videos—one with sign language only, one with captions only, and one with both sign language and captions—were made. This process employed an approved center for the deaf in Riyadh, Saudi Arabia, to ensure the sign language interpreter’s skill in interpreting SASL and the captions’ content accuracy. For the second stage, experts who work as associate professors at a large university in Saudi Arabia and have expertise and specialization in deaf education validated each of the copies.

#### Instruments

I used formative assessments and pretest/posttest throughout the entire study. In addition, I conducted interviews with a subset of the participant pool.

##### Formative assessments and pretest/posttest

I developed quizzes and final examinations for each experimental phase (declarative and procedural). Experts specializing in educational technology, computer education, and deaf education, whose qualifications are mentioned above, validated the quizzes and examinations to ensure the language level was appropriate for the deaf students’ reading level and the questions were appropriate to the videos’ content. The value of internal consistency analyses (Cronbach’s α coefficient) of all quizzes and examinations was from 0.84 to 0.93, which is acceptable according to Flora (2020) and Howell (2012).

##### Interview

Of the 54 participants, 15 were interviewed after the experiment to enhance the study’s results and to ascertain the participants’ attitudes toward the best method of illustration embedded in the video tutorials. I recorded the interviews so they could be transcribed later. The interview included three questions.

1. What difficulties did you face when you watched the videos?
2. Did the illustration method help you in acquiring new information?
3. Do you prefer this method of receiving new information on a new topic?

The official university sign language interpreter acted as the interpreter during the 15 interviews, asking each student the prepared questions using SASL. The students responded to the questions in SASL and the interpreter orally translated their signed responses for me. All of the students’ answers were then converted into a written text form and then encoded and categorized for statistical processing later. I used the *ad hoc* approach based on Munappy et al.’s (2020) recommendations for analyzing the interviews. I used this technique to discover the meanings and relationships that were important to the research from interviews. Each interview had a 10-min time limit.

#### Experimental procedure

Before the experiment, participants gathered in the college lab and received the written descriptive guide about the study. The same guide was presented *via* a video that employed SASL. The experiment was conducted in a computer lab equipped with computers and the Internet; each participant had a designated place to sit separately. All procedures were carried out in the presence of the official university sign language interpreter and myself to answer any questions and overcome any obstacles that might have occurred during the experiment.

**Figure 3:**
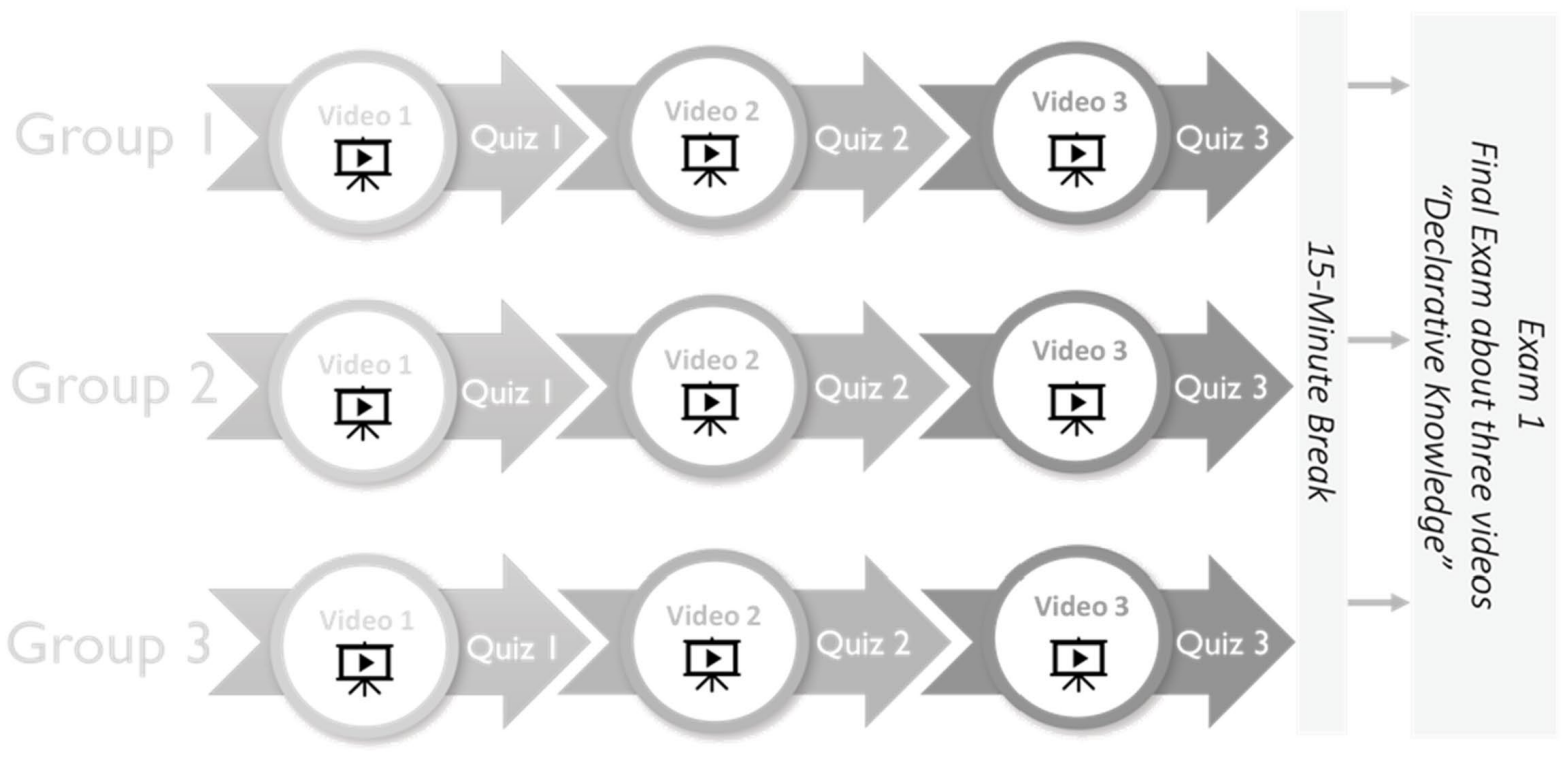
Procedure for experimental phase 1: Declarative knowledge.

As previously explained, phase 1 measured declarative knowledge and phase 2 measured procedural knowledge. The groups, again as previously described, consisted of Group S, who watched the videos that presented the information with only a sign language interpreter; Group C, who watched the videos that presented the information with only captions; and Group S&C, who watched the videos that presented the information with both an interpreter and captions. During the experiment’s declarative phase, the participants watched the first video (for their specific group) after which they went to a link to take a short quiz about the content they had just seen. Next, they watched the second video followed by another quiz link, and then to the third video, which was followed by another quiz link. Participants watched each video once and then took the respective quiz for that video. After completing all three video-quiz pairings, the participants took a 15-min break. Toward the end of the break, their screens notified the students that the experiment was about to resume, after which they were directed to the examination on declarative knowledge (see Figure 3).

**Figure 4:**
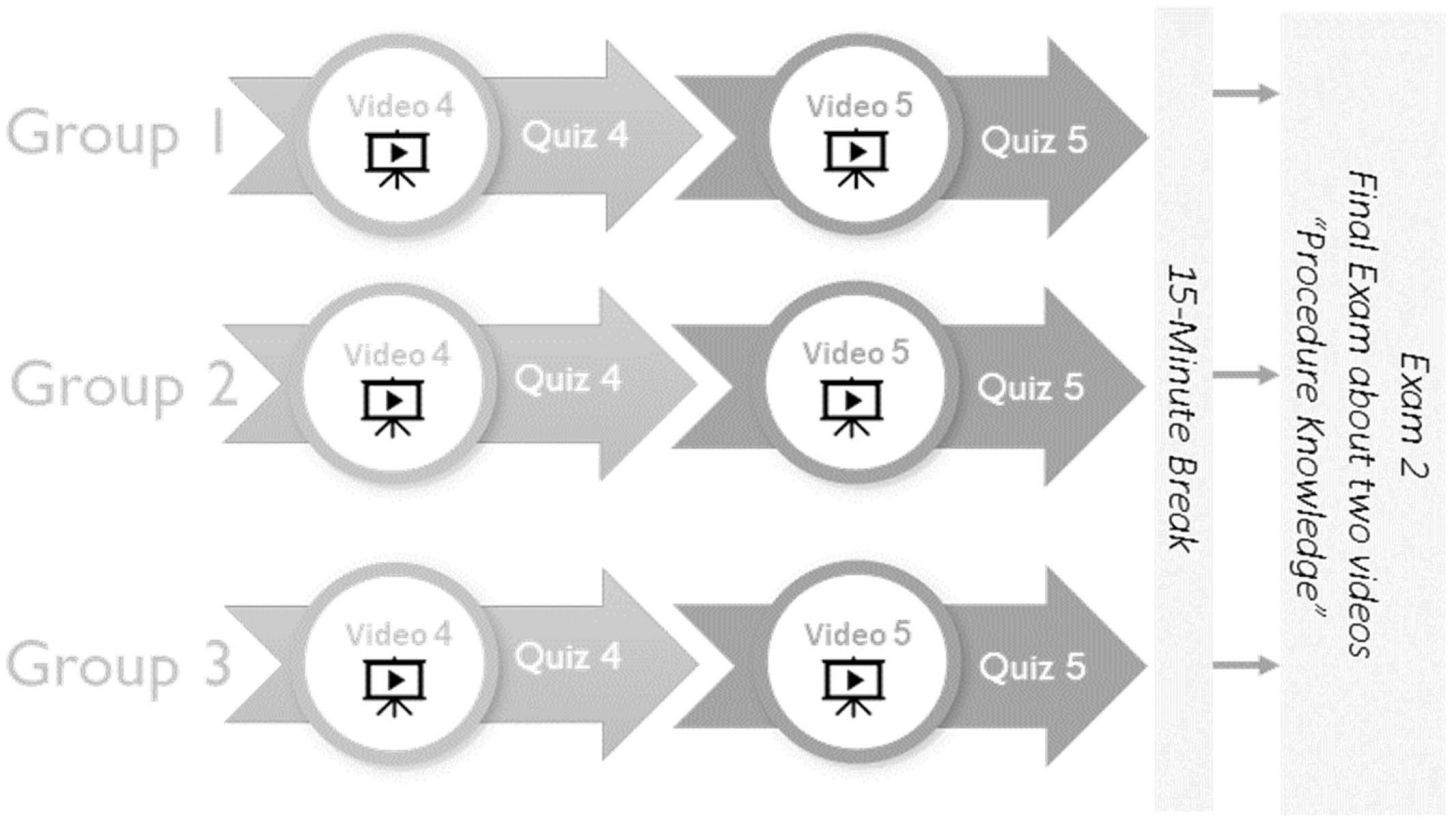
Procedure for experimental phase 2: Procedural knowledge.

After the experiment’s declarative phase, participants took a 30-min break. Next, they began phase 2, the experiment’s procedural phase. Similar to the process of phase 1, in the procedural phase, participants watched the fourth video for their respective group followed by a quiz, after which they were then directed to the fifth video and a quiz. After completing the two video-quiz pairings for this phase, the participants received a 15-min break. Following the break, the participants were then directed to the second examination, which was on procedural knowledge (see Figure 4).

The quizzes and examinations were collected electronically and were scored after all 54 participants had completed the study.

#### Measurement of variables

The study had two phases of experimentation. The dependent variable in the first phase was the deaf students’ acquisition of declarative knowledge based on their test-quiz scores. The independent variable in phase 1 was the accommodation, which comprised the three treatments: sign language interpreter alone (S), captioned text only (C), and sign language interpreter and captioned text combined (S&C). The second phase had one dependent variable, which was deaf students’ acquisition of procedural knowledge. The independent variable for phase 2 was again the accommodation, comprising the three different treatments of S, C, and S&C.

### Statistical analyses

Analyses were performed using IBM SPSS Statistics (Version 24). I preformed descriptive and internal consistency analyses (Cronbach’s α coefficient) while considering the limited number of participants. Levene’s test was utilized to calculate homogeneity of variance of groups, where, if *p* > 0.05, then it could be assumed that sample variances were approximately equal (Howell, 2012; Kim and Cribbie, 2018). One-way analysis of variance (ANOVA) was used to compare the participants’ quiz and final examination averages for each video. The comparison was conducted using the average of the three quizzes in phase 1 (based on a maximum possible averaged score of 25) and the average of the two quizzes in phase 2 (based on a maximum possible averaged score of 47.5) as the level 1 analysis; that is, the mean of the mean for the quizzes in both phases was calculated. Then, the level 2 analysis was based on the averages of the scores on the two exams (based on a top possible score of 50). To find significant directions, I used the recommended *post-hoc* test of Tukey’s honestly significant distance on the equal-group samples (Howell, 2012).

## Results

### Quantitative results

As stated at the beginning of this paper, the first research question addressed acquiring declarative knowledge:

Research question 1: Do the illustration methods of a sign language interpreter alone, captions alone, or both together differ significantly for increasing deaf students’ acquisition of declarative knowledge?

I calculated the quiz averages for the first three videos in phase 1 for each participant in each group, then I obtained the scores for the first exam, and I used one-way ANOVA between the three groups after ascertaining the value of homogeneity between groups for the averages of the three quizzes (Levene’s statistic = 2.686, *p* = 0.780 > 0.05) and the first examination (Levene’s statistic = 0.386, *p* = 0.682 > 0.05). Table 2 displays the means, standard deviations, and range of acquisition of declarative knowledge.

**Table 2:** Means, standard deviations, and range of acquisition of declarative knowledge for phase 1.

					95% CI for mean			
	*N*	Mean	Std. deviation	Std. error	Lower bound	Upper bound	Min	Max
**Three quizzes**								
Group S	18	20.19	1.800	0.424	19.29	21.08	18.33	23.33
Group C	18	23.61	1.428	0.196	22.90	24.32	21.67	25
Group S&C	18	17.32	0.832	0.196	16.90	17.73	16.67	18.33
**Exam 1**								
Group S	18	46.11	5.016	1.182	43.62	48.61	35	50
Group C	18	48.61	2.873	0.677	47.18	50.04	40	50
Group S&C	18	42.22	5.208	1.228	39.63	44.81	35	50

Dependent variable, Deaf students’ acquisition of declarative knowledge in phase 1; Independent variable, Accommodation comprising three treatments of sign language interpreter only (Group S), captions only (Group C), and sign language interpreter and captions combined (Group S&C). The score utilized for the analysis of the three quizzes is the average of the total of 75 points possible for these assessments, which is 25; the score utilized for the exam is 50.

Table 2 reveals the statistically significant differences among the three groups on the declarative content quizzes and the examination [*F*(2, 53) = 89.805, *p* < 0.001, η^2^ = 0.779] and [*F*(2, 53) = 9.246, *p* < 0.001, η^2^ = 0.266], respectively. Tukey’s *post-hoc* tests revealed statistically significant differences in quiz averages in favor of Group C over Groups S and S &C, and in favor of Group S &C over Group S (*p* < 0.001). The analysis of the exam scores revealed statistically significant differences for Group S over Group S&C (*p* = 0.032 < 0.05), and for Group C over Group S&C (*p* < 0.001), but no statistically significant differences were revealed between Groups S and C (*p* = 0.227 > 0.05). Based on the students’ scores in the quizzes in the presentation of declarative knowledge *via* video tutorial, the results indicated that the use of captions only was more effective when the participants were assessed immediately after watching the videos. Furthermore, based on their exam 1 scores, both captions only and sign language only had the same effect when the participants were assessed.

The second research question involved acquiring procedural knowledge.

Research question 2: Do the illustration methods of a sign language interpreter alone, captions alone, or both together differ significantly for increasing deaf students’ acquisition of procedural knowledge?

I calculated quiz averages for the two videos in phase 2 for each participant in each group, then I obtained the scores for the second examination, and I used one-way ANOVA between the three groups. The value of homogeneity was ascertained among the groups for the averages of the two quizzes (Levene’s statistic = 1.325, *p* = 0.408 > 0.05) and for the examination (Levene’s statistic = 0.051, *p* = 0.950 > 0.05). Table 3 displays the means, standard deviations, and range of acquisition of procedural knowledge.

**Table 3:** Means, standard deviations, and range of acquisition of procedural knowledge for phase 2.

					95% CI for mean			
	*N*	Mean	Std. deviation	Std. error	Lower bound	Upper bound	Min	Max
**Two quizzes**								
Group S	18	43.19	2.819	0.664	41.79	44.60	37.5	47.5
Group C	18	38.75	3.457	0.815	37.03	40.47	30	45
Group S&C	18	34.72	3.627	0.855	32.92	36.53	30	42.5
**Exam 2**								
Group S	18	45.00	3.835	0.904	43.09	46.91	40	50
Group C	18	39.44	3.792	0.894	37.56	41.33	30	45
Group S&C	18	36.11	3.234	0.762	34.50	37.72	30	40

Dependent variable, Deaf students’ acquisition of procedural knowledge in phase 2. Independent variable, Accommodation comprising three treatments: (a) sign language interpreter only (Group S), (b) captions only (Group C), and (c) sign language interpreter and captions combined (Group S&C). The score utilized for the analysis of the two quizzes is the average of the total of 95 points possible for these assessments, which is 47.5; the score utilized for the exam is 50.

Table 3 reveals the statistically significant group differences among averages of the procedural quizzes and examination [*F*(2, 53) = 29.345, *p* < 0.001, η^2^ = 0.535] and [*F*(2, 53) = 27.537, *p* < 0.001, η^2^ = 0.519], respectively. Tukey’s honestly significant distance test revealed statistically significant differences in quiz averages in favor of Group S over Groups C and S&C and in favor of Group C over Group S&C (*p* < 0.001). The procedural examination showed statistically significant differences in Group S over Groups C and S&C (*p* < 0.001) and in Group C over Group S&C (*p* = 0.22 < 0.050). These results indicate that using a sign language interpreter only in the video was more effective—based on the participants’ scores—in presenting procedural content, followed by captions only. Simultaneous captions and signed interpretation—based on the participants’ scores—was the least effective. The findings were the same whether participants were assessed immediately after watching the videos *via* the quizzes or after a while *via* exam 2.

### Qualitative results

As previously described, I conducted 10-min personal interviews with 15 of the 54 participants after I completed the quantitative data collection to expand on and further confirm the study results. During the individual interviews, the university sign language interpreter asked each student the prepared questions using SASL, to which the students responded in SASL; the interpreter then immediately verbally translated each student’s signed responses for me. I recorded the interviews to analyze the content later.

The process for analyzing and reporting on the qualitative section of the study was as follows. First, the data were collected through the 15 interviews after which I stored and labeled them. Next, using the interview recordings, I transcribed the interpreter’s translations of the students’ answers. These data were then classified and coded to be grouped based on similarities. Then, I characterized the qualitative data, calculated the frequencies, and identified the themes that recurred across the interviews. Finally, I created a description of the codes to identify the links between the themes and any patterns that existed (Creswell, 2014). The following describes the findings obtained from the interviews.

I asked three questions during the interviews. Regarding the first question (“What are the difficulties, you faced when you watched the videos?”), most of the participants indicated that learning through video was easy and enjoyable and that the illustrations included within the study videos supported their understanding of the information provided. Regarding the second question (“Did the illustration method help you in acquiring new information?”), the group that watched the video with sign language indicated that the sign language interpreter helped them to understand the presented content easily, especially because they were proficient in sign language and were familiar with it. The group that watched the video with captions also indicated that the captioning helped them understand the information easily, but that they found it difficult to understand some of the procedural information, whereas it was easier for them to understand the declarative information with the captioning. Regarding the third question (“Do you prefer this method of receiving new information on a new topic?”), all participants expressed their enthusiasm for learning using the video method, again with the use of a sign language interpreter to facilitate access to information. They also all felt that learning *via* video was better than learning *via* traditional face-to-face methods and that they preferred it for acquiring new information.

## Discussion

It is important to identify the most appropriate illustration method—or methods—that can be embedded in video instructional materials that best facilitates acquiring declarative knowledge and procedural knowledge, especially when the topic of learning relates to digital technology. Therefore, this study examined the effects of three illustration methods embedded in video tutorials—sign language interpreter only, captions only, and both captions and a sign language interpreter—on deaf students’ comprehension of declarative and procedural knowledge of topics in digital technology. Students’ knowledge on both short quizzes and longer examinations after viewing the tutorial videos presented in each of the three methods was assessed, and further insight was obtained through the interviews. Evidence emerged regarding the most appropriate means of illustration through the results and interviews, which I present in the following sections.

### Individual examination of study phases

#### Phase 1: Declarative knowledge

The results of the first phase, which investigated comprehension of declarative content, found that the quiz results favored using only captions over the other two illustration methods. As noted in the results, I determined this using Tukey’s honestly significant distance test, which revealed statistically significant differences in quiz averages in favor of Group C (only captions) over Group S (only sign) and Group S&C (both sign and captions; *p* < 0.001). These results are congruent with the findings of past studies that indicated that adding only captions to videos increased the deaf students’ comprehension (e.g., Debevc et al., 2015; Smith et al., 2017; Al-Ibrahim, 2019). This also complements Alsalamah’s (2020) finding that captions had a greater positive effect on deaf students’ comprehension than did including an interpreter in video and the services of a note taker.

According to the analysis of the quiz results, synchronous use of a sign language interpreter and captions was the least effective method for declarative content on topics of digital technology, which is similar to the findings of others students (e.g., Yoon and Kim, 2011; Debevc et al., 2015). Other studies such as that of Alexander et al. (2017), has asserted that when both captions and a sign language interpreter are included, the order in which the captions and sign language interpretations are displayed needs consideration. However, unlike the results of Alexander et al. (2017) study, the scope of the present study did not include varying the order but rather displayed the captions and the sign language simultaneously. However, per the students’ scores on the examinations, simultaneous captions and sign language interpretation was second in effectiveness on the illustration of content both by sign language only and by captions—because the latter two methods had an equally positive effect on students’ acquisition of declarative content. During the interviews, the 15 deaf students confirmed that the videos with only captions and the videos with only the sign language interpreter helped them to absorb the declarative information presented, but some indicated that the combination of the two methods created a type of mental distraction. This could be due to how the eye had difficulty following three visuals at the same time, in this case the captions, sign language, and the displayed video that contained animation and images (Mayer, 2009; Smith et al., 2017). In addition, declarative information is sometimes presented in the form of texts that students are accustomed to reading in books or through videos; therefore, it was easier for them to learn *via* the videos that had the captions only included within the video compared to other methods of illustration because of its familiarity.

#### Phase 2: Procedural knowledge

In phase 2, which investigated the comprehension of procedural knowledge, the findings regarding effectiveness of delivery method were the same from both the quizzes and the examinations. The personal interviews with the participants confirmed these findings. Using only a sign language interpreter had the greatest positive effect on deaf students’ comprehension followed by captions only. The videos with simultaneous captions and signed interpretation of the procedural content were the least effective. These findings agree with those of studies that demonstrated a positive effect on comprehension of procedural content about cardiopulmonary resuscitation (Galindo-Neto et al., 2019) and on oral hygiene (Fageeh and Mansoor, 2020). However, these studies employed only a sign language interpreter. In addition, this confirms Hashim and Tasir’s (2020) finding that deaf students showed a preference for video with a sign language interpreter.

Contrary to some research, the present study revealed that including captions and a sign language interpreter synchronously had a lower positive effect on comprehension of procedural knowledge than other methods did (sign only and captions only). For example, Debevc et al. (2015) and Yoon and Kim (2011) found that using each method separately was less effective for comprehension than their use simultaneously was. Considering deaf students communicate with their teachers and with each other through sign language, the delivery of procedural information that requires specific steps is easier for them to learn and understand through presentations that use sign language because they have more familiarity with this method than they do with others.

Multiple factors contributed to the present study’s findings that are specific to the Saudi Arabia context. First, although deaf people in Saudi are increasing their familiarity with SASL vocabulary and with the use of technology, and although the majority of signs in SASL share vocabulary and linguistic features with AS (Alamari, 2017), most teachers of deaf students in Saudi have poorly developed SASL skills, as Alofi et al. (2019) noted. Moreover, in Saudi Arabia, deaf students are exposed to three languages simultaneously: AS, SASL, and the slang form of signed Arabic that incorporates vocabularies and linguistic structures different from AS and SASL (Alsalem, 2018). These challenges the Saudi Deaf community faces are only compounded by the fact that SASL interpreting in Saudi Arabia is still an emerging profession (Almotiri, 2017). As a result, skilled SASL interpreters available for deaf students at the college level are lacking (Alofi et al., 2019). Given all these factors, for a video tutorial presenting rich declarative content, using sign language only might not be the optimal presentation method.

Additionally, although using only captions plus supplemental instructions in a video tutorial is effective for college-level deaf students, as Alsalamah (2020) demonstrated, for captions to be effective, it might first be necessary to support and improve deaf students’ reading comprehension (Beal-Alvarez and Cannon, 2014).

The present study’s findings appear consistent with principles of the cognitive theory of multimedia learning, which addresses the importance of minimizing sources of external cognitive (over)load that might result from presenting both captions and sign language simultaneously (Mayer, 2009; Smith et al., 2017). Furthermore, researchers did not clarify the type of content they were assessing the comprehension of in previous studies.

When comparing the use of only captions and the simultaneous use of both an interpreter and captions, this study’s results found that only captions were more effective with declarative knowledge. As previously mentioned, declarative knowledge usually includes more concepts and information than procedural knowledge does; therefore, sign language alone might not be adequate for delivering declarative content. Yoon and Kim’s (2011) work supports this finding, which shows that adding a sign language interpreter to a video in combination with captions helps ameliorate how deaf students as a population tend to lag behind their hearing peers in reading comprehension (Bickham, 2015). This could explain the last of this study’s findings, wherein the use of both sign language and captions were better for students’ acquisition of declarative content, which, as Alsalamah (2020) stated, in most cases requires a higher level of reading skill than does procedural content. Similar to those of other studies, the present research confirms that using a sign-language interpreter does not adversely affect comprehension when teaching procedural content presented in a video tutorial (Galindo-Neto et al., 2019; Fageeh and Mansoor, 2020).

### Implications and conclusions

With the rapid changes in the use of technology around the world in general and specifically in Saudi Arabia, it is essential to utilize digital technology’s capabilities to facilitate both learning and teaching processes. Currently, the Ministry of Education in Saudi Arabia is working on an initiative to convert the content of textbooks into interactive books. Therefore, this study’s findings contribute to assisting decision-makers in choosing the appropriate video tutorial delivery methods for deaf students in the field of digital technology, which the Saudi Ministry of Education has introduced and will implement as a new curriculum beginning in 2023 for students from Grades 4–12.

This study found that some illustration methods are more effective for certain types of knowledge, which is precisely what the study sought to discover. Specifically, the present study’s results recommend using captioned delivery for teaching declarative content and using a sign language interpreter for teaching procedural content. Therefore, instructional designers and educational practitioners in the field of deaf education should consider the type of content and subject area when deciding the appropriate video delivery method for deaf students.

### Limitations

The present study possessed certain limitations. First, the research sample was small; therefore, the results cannot be generalized. Second, the field of examination was related to digital technology, thus, the results might differ for other areas and topics. Third, the participants of the study varied regarding socioeconomic and cultural backgrounds from within Saudi Arabia and no specific tests were conducted to measure how these differences could affect the study results. Finally, the study did not employ a control group that learned the content *via* a traditional delivery method, such as in the classroom setting, rather than *via* video, which might have strengthened the study by providing further insight into the effectiveness of video as a means of instruction when compared to traditional methods.

### Future recommendations

Recommendations for future studies regarding the most effective methods to present instructional content in digital technology to deaf students include assessing the retention of content delivered in this way over time. The present study only measured the students’ comprehension almost immediately after they had watched the videos; therefore, further study is required to measure the retention of the learned content after various intervals. Future research should also measure student satisfaction and motivation for each method and setting.

Moreover, future studies should examine the effect of different methods of content presentation for deaf students in other settings, for example, in kindergarten through twelfth grade and in face-to-face learning environments. For example, future studies could compare the effectiveness of open-and closed-captioning, signed only, and signing plus open and closed captioning, all of which would enhance instructional designers’ knowledge of the learning preferences of deaf students in kindergarten through twelfth grade settings regarding digital technology use. In addition, investigating an instructor’s use of supplemental instruction along with or following the students viewing the video tutorials may increase students’ retention and application of the content presented in the videos (Alsalamah, 2020). Incorporating the tutorial videos as described in the present study within a further interactive and problem-solving pedagogy—including discussion forums, notes, and a glossary of terms—presented *via* sign language videos as Hashim and Tasir (2020) described would enable deaf students to access and use the content presented in e-learning formats with greater choice relevant to their learning preferences.

Replicating the present study and adding animations and avatars as well as captions and signing would provide opportunities to investigate ways these video techniques can facilitate deaf students’ acquisition of content in developing both their computer literacy skills and their computer programming skills.

## Data availability statement

The raw data supporting the conclusions of this article will be made available by the author, without undue reservation.

## Ethics statement

The studies involving human participants were reviewed and approved by Deaf Higher Education Ethics Committee at King Saud University. The patients/participants provided their written informed consent to participate in this study.

## Author contributions

The author confirms being the sole contributor of this work and has approved it for publication.

## Conflict of interest

The author declares that the research was conducted in the absence of any commercial or financial relationships that could be construed as a potential conflict of interest.

## Publisher’s note

All claims expressed in this article are solely those of the authors and do not necessarily represent those of their affiliated organizations, or those of the publisher, the editors and the reviewers. Any product that may be evaluated in this article, or claim that may be made by its manufacturer, is not guaranteed or endorsed by the publisher.
